# Strain amplitude sweep testing of oscillatory shear of transient networks with controlled network structures

**DOI:** 10.1080/14686996.2026.2613918

**Published:** 2026-01-12

**Authors:** Ren Sato, Yoshifumi Yamagata, Moe Araida, Taisuke Sato, Mitsuru Naito, Hiroshi Sekiguchi, Keishi Akada, Ung-Il Chung, Takuya Katashima

**Affiliations:** a Department of Bioengineering, Graduate School of Engineering, The University of Tokyo, Bunkyo-Ku, Tokyo, Japan; b Anton Paar Japan K.K. Riverside Sumida 1st Fl, Sumida-Ku, Tokyo, Japan; c Research Institute for Science and Technology, Tokyo University of Science, Noda-Shi, Chiba, Japan; d Photonic Lattice Inc. LABO CITY SENDAI, Sendai-City, Japan; e Department of Materials Science and Technology, Faculty of Advanced Engineering, Tokyo University of Science, Katsushika-Ku, Tokyo, Japan; f Japan Synchrotron Radiation Research Institute, Sayo-Gun, Hyogo, Japan; g Center for Disease Biology and Integrative Medicine, Graduate School of Medicine, The University of Tokyo, Bunkyo-Ku, Tokyo, Japan

**Keywords:** Rheology, transient network, viscoelasticity, nonlinear, LAOS

## Abstract

The origin of nonlinear stress behaviors in transient networks during oscillatory shear measurements is investigated in this study via two-dimensional rheo-optics observations of a systematically controlled model system comprising tetra-armed polyethylene glycols (Tetra-PEG slime). Transient networks are characterized by their temporary crosslinks. However, the strain scale at which nonlinear viscoelastic responses begin to emerge under strain amplitude sweep in oscillatory shear deformations remains poorly characterized. The uncertainty of nonlinear onset can be attributed, at least in part, to the heterogeneous structures inherent to conventional transient networks, as well as to the limited availability of detailed experimental evaluations. We overcome these limitations by employing Tetra-PEG slime, which possesses a well-defined network structures with uniform strand lengths and functionalities. Rheo-polarization imaging reveals homogeneous deformation in the linear regime and a scaling change of retardation – strain relation around the onset of nonlinearity. Meanwhile, Rheo-SAXS measurements confirmed the absence of nanoscale structural changes. The elastic contribution per network strand at the critical strain (*W*_c,0_) approximately collapses onto a single master curve when plotted against the Weissenberg number (Wi_max_), following the relation *W*_c,0_ ∝ Wi_max_^2^. This scaling suggests that the onset of nonlinearity is governed by the balance between molecular relaxation and applied deformation. These findings provide molecular-level insights into the nonlinear elasticity of transient networks and offer a framework to design soft materials with tunable nonlinear responses.

## IMPACT STATEMENT

This paper provides molecular-level insight into onset of nonlinear viscoelasticity of transient networks, revealing scaling of elastic contribution under strain amplitude sweep oscillatory shear using well-defined model systems.

## GRAPHICAL ABSTRACT

**Figure:**
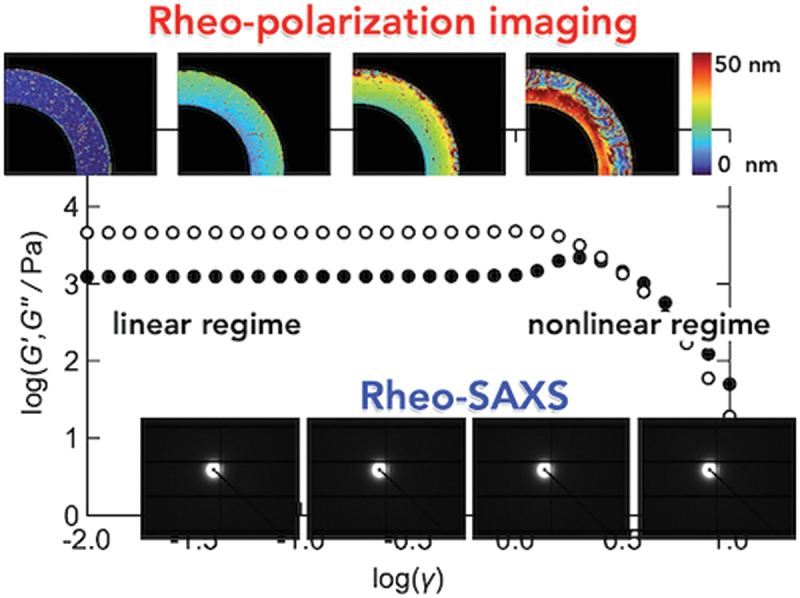

## Introduction

Transient networks such as threadlike micelles [1–4] or associative polymers [5–9] are a class of viscoelastic liquid materials with three-dimensional network structures formed through reversible interactions (*i.e*., coordination, hydrogen, and dynamic covalent bonds). The viscoelasticity of these materials is effectively characterized using small-amplitude oscillatory shear (SAOS). In SAOS measurements, a sinusoidal strain *γ*(*t*) = *γ*_0_ sin(*ωt*) is applied to the sample, where *γ*_0_ and *ω* are the strain amplitude and the angular frequency, respectively, while *t* represents the time. The corresponding stress response is given by *σ*(*t*) = *σ*_0_ sin(*ωt* +*δ*), where *δ* is the loss phase angle and *σ*_0_ represents the stress amplitude. In this framework, the strain and stress are treated as the imposed input and measured output, respectively.

When the strain amplitude is small, *σ*_0_ is proportional to *γ*_0_, exhibiting linear viscoelasticity, which reflects the molecular dynamics and structures at the equilibrium state. According to previous studies, the SAOS behavior of transient networks is often described by the Maxwell model, which indicates that stress relaxation is governed by a single relaxation mode [1,6,10]. As *γ*_0_ increases beyond a certain threshold, *σ*_0_ deviates from a proportional relationship with *γ*_0_, suggesting the onset of nonlinear viscoelastic behavior. We treat the onset strain phenomenologically as an experimental indicator of nonlinearity. These nonlinear features are interpreted as the complex interplay between bond dissociation – reformation dynamics and polymer chain deformation [11,12].

Thus far, steady shear flow measurements have been employed to probe such nonlinear rheological behavior, providing information on the viscosity and stress response as a function of deformation rate under steady-state conditions. However, when deformation is imposed on a time scale shorter than the terminal relaxation time of the material, the system may not reach a steady state. Further, steady shear tests provide limited insight into microstructural evolution or relaxation timescales because the measurements reflect time-averaged responses [13–15]. Nonlinear responses in complex fluids, however, are governed by both the magnitude of the imposed strain and the strain rate, which cannot be varied independently in conventional steady or transient shear protocols. In contrast, large-amplitude oscillatory shear (LAOS) allows independent control of the strain amplitude (*γ*_0_) and angular frequency (*ω*) [16–20], providing a framework to identify and map the onset of the nonlinear regime under time-dependent deformation. In addition, LAOS provides access to rich harmonic information regarding the stress signal, which can be analyzed using Fourier-transform rheology [19,21–24] or Chebyshev polynomial decomposition [25–28] to quantify distinct elastic and viscous nonlinearities. For an arbitrary *γ*_0_, the stress response can be expressed as a harmonic series,

(1) $$\begin{array}{c} \sigma \left(t\right)=\underset{n=\mathrm{odd}}{\sum }\left[\sigma_{n}^{\prime }\left(\omega ,\gamma_{0}\right)\sin\left(\mathrm{n\omega t}\right)+\sigma_{n}^{{\;}^{\prime \prime }}\left(\omega ,\gamma_{0}\right)\cos\left(\mathrm{n\omega t}\right)\right] \end{array}$$

A convenient scalar measure of the emergence of nonlinearity in Fourier-transform rheology is the normalized third-harmonic intensity, *I*_3_/*I*_1_ (often equivalently reported as ∣*σ*_3_∣/∣*σ*_1_∣), which is approximately zero in the linear regime and increases as higher harmonics develop with increasing *γ*_0_. In the Chebyshev framework, the stress is decomposed into elastic and viscous contributions parameterized by the normalized strain and strain rate, and expanded in orthogonal Chebyshev polynomials of the first kind *T_n_*(⋅) as

$$\sigma \left(t\right)=\underset{n=\mathrm{odd}}{\sum }\left[\gamma_{0}e_{n}\left(\omega ,\gamma_{0}\right)T_{n}\left(x\right)+\gamma_{0}\omega v_{n}\left(\omega ,\gamma_{0}\right)T_{n}\left(y\right)\right]$$

(2) $$\begin{array}{c} x\left(t\right)=\frac{\gamma \left(t\right)}{\gamma_{0}}=\sin\left(\mathrm{\omega t}\right),y\left(t\right)=\frac{\dot{\gamma }\left(t\right)}{\gamma_{0}\omega }=\cos\left(\mathrm{\omega t}\right) \end{array}$$

The normalized third-order elastic and viscous nonlinearities are commonly reported as *e*_3_/*e*_1_ and *v*_3_/*v*_1_, which quantify intra-cycle elastic and viscous distortions relative to the fundamental Chebyshev modes, respectively. Therefore, nonlinear contributions evolve continuously with *γ*_0_ and do not imply a mathematically sharp critical strain or stress. For practical comparison across conditions, we therefore introduce an operational characteristic strain and stress (*γ*_c_, *σ*_c_), defined phenomenologically.

Combined with optical methods such as birefringence or scattering, strain amplitude sweep experiments can correlate macroscopic stress responses with microstructural and molecular orientation dynamics [29–34]. However, a complete understanding of the nonlinear viscoelastic response of transient networks has been difficult to achieve, partly because the structural and dynamic heterogeneities of conventional systems make it challenging to establish quantitative correlations between microscopic structure and macroscopic rheology.

Conventional transient networks possess structural heterogeneities arising from both static and dynamic origins [35–38]. Static heterogeneity refers to structural defects at the equilibrium state, such as dangling chains, loop defects, and the polydispersity of strand lengths and crosslinking functionalities. In contrast, dynamic heterogeneity arises from the coexistence of slow and fast relaxation modes, which reflects complex interactions among network chains, unimers, and micellar aggregates. These hierarchical and uncontrolled structures lead to complex relaxation processes, which obscure the direct correlation between molecular dynamics and macroscopic stress responses under nonlinear deformation. Consequently, a molecular-level understanding of rheological behavior in transient networks has remained elusive because of the intrinsic difficulty in controlling or quantitatively assessing these heterogeneities.

To address these challenges, we recently developed a model transient network system with a well-controlled structure referred to as Tetra-PEG slime [39–41]. This system includes two tetra-armed polyethylene glycol (PEG) precursors end-functionalized with phenylboronic acid and diol groups, which form dynamic covalent bonds through reversible boronate ester linkages. The narrow molecular weight distribution and symmetric network architecture ensure uniform strand lengths and mobilities, effectively minimizing static and dynamic heterogeneities. This molecular precision eliminates uncontrolled hierarchical structures, directly investigating the correlation between reversible bond dynamics and nonlinear mechanical responses under deformation. It should be noted that, even when molecular weight and polymer concentration are systematically varied in conventional systems, quantitative control over network connectivity and the strand-length distribution is generally nontrivial. Establishing a controlled network is therefore an essential first step toward identifying potential molecular-level origins of the onset of nonlinearity in transient networks, while keeping the discussion anchored to experimentally accessible observables.

Nonlinear viscoelastic responses evolve continuously with increasing strain amplitude, and thus the ‘onset of nonlinearity’ does not correspond to a mathematically sharp transition. However, when comparing materials or compositions, an operational onset scale – defined through reproducible experimental criteria – is essential to distinguish the strain range wherein linear viscoelasticity begins to break down. In this study, we focus on identifying and comparing such onset strain scales across structurally controlled transient networks, rather than characterizing the fully developed nonlinear regimes. This framing enables us to correlate the emergence of nonlinear elastic behavior with molecular relaxation processes while avoiding ambiguities associated with strongly nonlinear flow instabilities. To discuss the molecular background, we utilized rheo-polarization imaging (PI) [42,43] and rheo-small-angle X-ray scattering (SAXS) [44–50]. These results offer valuable insights into the onset of nonlinear behavior under oscillatory strain sweep in transient networks.

## Materials and methods

### Sample preparation

Tetra-armed polyethylene glycol (tetra-PEG) whose end groups were modified with 4-carboxy-3-fluorophenylboronic acid (FPBA) (tetra-PEG-FPBA, *M*_w_ = 10, 20, and 40 kg mol^−1^) or glucono-δ-lactone (GDL) (tetra-PEG-GDL, *M*_w_ = 10, 20, and 40 kg mol^−1^) were purchased from XIAMEN SINOPEG BIOTECH Co., Ltd. (China). Tetra-PEG-FPBA and tetra-PEG-GDL were dissolved separately in a phosphate buffer (pH 7.4, 200 mM) at mass concentrations of 40, 60, 80, and 100 g L^−1^. These solutions were then mixed at equal volume to obtain the Tetra-PEG slime.

### Oscillatory shear measurement

**Figure 1.**
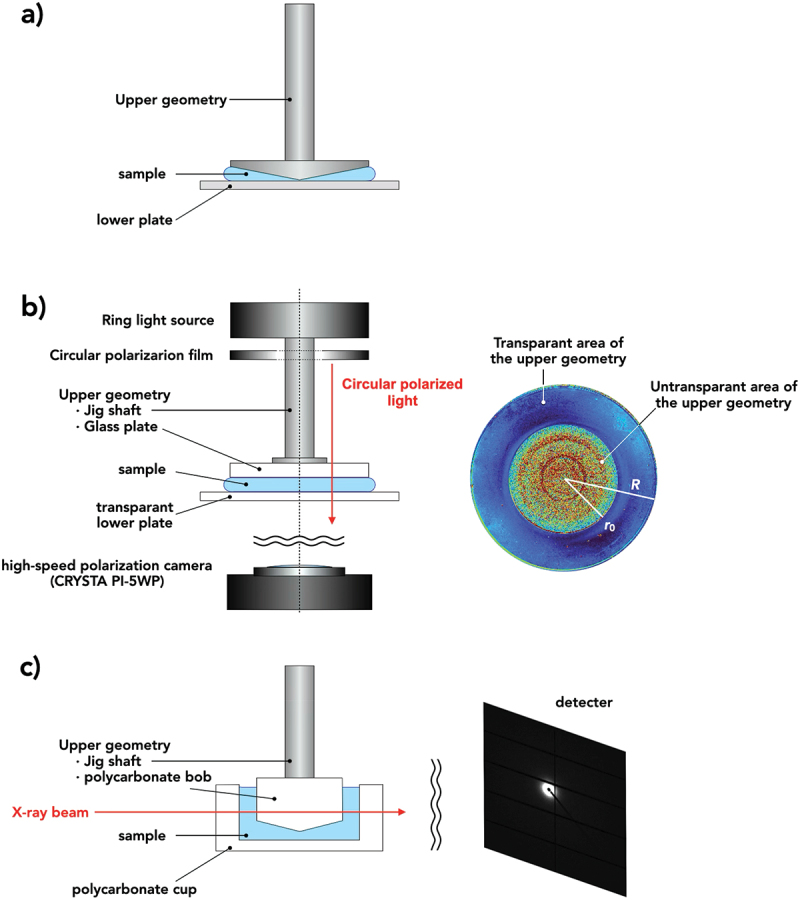
Schematic illustration of experimental setups for small-amplitude oscillatory shear (SAOS) and strain amplitude strain measurements (a), Rheo-polarization imaging measurements (b), and Rheo-SAXS measurements (c). *R* ( = 21.5 mm) indicates the radius of the upper glass plate and *r*_0_ ( = 14.5 mm) is the radius of the non-transparent area of the upper geometry.

Oscillatory shear measurements were conducted at 25°C using a stress-controlled rheometer (MCR302, Anton Paar, Austria) equipped with a cone-plate fixture, in which a sinusoidal strain was imposed by feedback control of the applied stress. The cone had a diameter of 25 mm and cone angle of 4°. For small amplitude oscillatory shear (SAOS) measurements, the angular frequency dependences (0.01–10 rad s^−1^) of the storage (*G'*) and loss (*G''*) moduli were measured at a strain amplitude of 0.01, which was confirmed to be within linear viscoelasticity. For strain amplitude sweep measurements, the strain amplitude dependences (0.01–10) of the storage (*G'*) and loss (*G''*) moduli were measured at angular frequencies of 0.1, 1.0, and 10 rad s^−1^. The schematic illustration of the experimental setup is shown in Figure 1(a).

### Rheo-polarization imaging measurement

The same rheometer equipped with a transparent glass parallel plates (diameter: 43 mm) as the upper geometry was used for PI during the strain amplitude sweep measurements. The sample thickness was fixed at 2 mm for all measurements. The rheometer head featured a lighting system that comprised a ring white LED and circular polarizing film to project circularly polarized light onto the sample. A high-speed polarization camera (CRYSTA PI-5WP; Photonic Lattice Inc., Japan) was positioned beneath the lower plate, and a band-pass filter with a transmission spectrum centered at 543 nm was placed in front of the camera. This setup enabled real-time observations of the retardation (birefringence) and orientation axis distributions. This camera captured 12-bit grayscale intensity images (848 × 680 pixels) and detected the light intensity passing through a photonic crystal element that functioned as a rotational linear polarizer. Four linear polarizers were set at 45° intervals, and their outputs were integrated into four neighboring pixels of the image sensor. The schematic illustration of the experimental setup is shown in Figure 1(b). Although the rheo-polarimetry module is available only with a parallel-plate (PP) fixture, only optical retardation is analyzed to assess spatial deformation, and PP-derived stress data were not used for quantitative comparison. Because the PP geometry intrinsically exhibits a radial strain gradient, retardation was evaluated as a function of the local strain amplitude after correcting for the radial position *r*. Stress-based comparisons across geometries are restricted to the regime *γ*_0_ ≤ *γ*_c_, wherein flow remains homogeneous, and stress responses agree across geometries.

### Rheo-SAXS measurement

Rheo-SAXS measurements under strain amplitude sweep were performed using the same rheometer equipped with a polycarbonate coaxial double-cylinder geometry (bob and cup radii = 19.5 and 20.5 mm, respectively). The rheometer was integrated into a synchrotron SAXS setup at beamline BL40XU (SPring-8, Japan). A monochromatic X-ray beam with a wavelength of 1.24 × 10^−10^ m was used, and the range of the provided scattering vector (*q*) was 2.76 × 10^−2^–1.70 nm^−1^. Scattering patterns were recorded using the two-dimensional detector PILATUS1M. The strain amplitude sweep experiments were conducted at strain amplitudes of *γ*_0_ = 0.01–2 and an angular frequency of 10 rad s^−1^. In our Rheo-SAXS setup, precise synchronization between the oscillatory motion of the rheometer and the detector time-slicing was not available. The X-ray exposure time was 3.142 s, corresponding to five oscillation cycles during which SAXS patterns were collected, so that all phases of the oscillation were averaged while avoiding beam-induced damage to the shearing cell. An X-ray beam passed through the center of the polycarbonate Couette cell, perpendicular to its rotational axis. The schematic illustration of the experimental setup is shown in Figure 1c. We verified that strain amplitude sweep measurements performed with cone-plate (CP) and concentric-cylinder (CC) fixtures yielded consistent stress – strain responses, waveforms, and Lissajous curves within the strain regime below the onset of nonlinearity (*γ* ≤ *γ*_c_). Accordingly, all quantitative analyzes in this work are based on CP-derived stress data within *γ* ≤ *γ*_c_, and CC-derived stress data are used only in conjunction with Rheo-SAXS under the same strain limitations.

## Results and discussion

**Figure 2.**
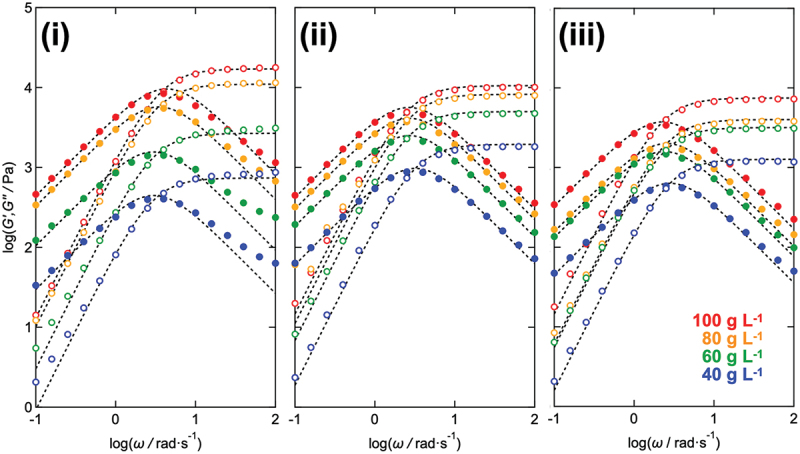
Angular frequency (*ω*) dependence of the storage (*G’*, open circles) and loss (*G”*, filled circles) moduli of the Tetra-PEG slimes (i: *M*_*w*_ = 10 kg mol^−1^, ii: *M*_*w*_ = 20 kg mol^−1^, iii: *M*_*w*_ = 40 kg mol^−1^) with various polymer concentrations (*c*). The dashed curves represent the fitting results obtained using the Maxwell model.

Figure 2 shows the representative SAOS measurement results for Tetra-PEG slime samples with various molecular weights and polymer concentrations. In the viscoelastic spectrum, the storage modulus *G'* plateaued at high frequencies, but exhibited terminal relaxation behavior (*G'* ~ *ω*^2^ and *G''* ~ *ω*) at low frequencies; within this region, the loss modulus *G'* formed a roughly symmetric peak around its maximum. These frequency dependences of the storage and loss moduli of the Tetra-PEG slime were quantitatively described well by a Maxwell model, which is expressed as.

(3) $$\begin{array}{c} G{\;}^{\prime }=\Delta G\frac{\omega^{2}\tau^{2}}{1+\omega^{2}\tau^{2}},G^{{\;}^{\prime \prime }}=\mathrm{\Delta G}\frac{\omega \tau }{1+\omega^{2}\tau^{2}} \end{array}$$

where Δ*G* and *τ* represent the shear modulus and relaxation time, respectively. The Δ*G* and *τ* values for each sample are summarized in Table 1. As indicated by the dashed fitting lines, the Maxwell-model fit captures these features well over the fitted frequency range. Notably, in the high-frequency regions of the low-polymer-concentration samples, modes associated with the PEG chains were observed. A more detailed molecular interpretation of the relaxation process, which we have discussed previously, is beyond the scope of the present study [40].

**Table 1.** Modulus (*ΔG*, top) and relaxation time (*τ*, bottom) of the Tetra-PEG slime with various molecular weight (*M*_w_) and polymer concentration (*c*), as estimated by the Maxwell model.

*ΔG* /Pa			*M*_w_ [kg mol^−1^]	
		10	20	40
*c* [g L^−1^]	100	1.7 × 10^4^	1.1 × 10^4^	7.4 × 10^3^
	80	1.2 × 10^4^	7.8 × 10^3^	4.0 × 10^3^
	60	3.1 × 10^3^	4.8 × 10^3^	3.1 × 10^3^
	40	8.9 × 10^2^	1.9 × 10^3^	1.3 × 10^3^
*τ*/s			*M*_w_ [kg mol^−1^]	
		10	20	40
*c* [g L^−1^]	100	2.6 × 10^−1^	4.1 × 10^−1^	4.5 × 10^−1^
	80	2.9 × 10^−1^	4.0 × 10^−1^	4.0 × 10^−1^
	60	3.4 × 10^−1^	3.9 × 10^−1^	4.2 × 10^−1^
	40	3.4 × 10^−1^	3.3 × 10^−1^	3.6 × 10^−1^

**Figure 3.**
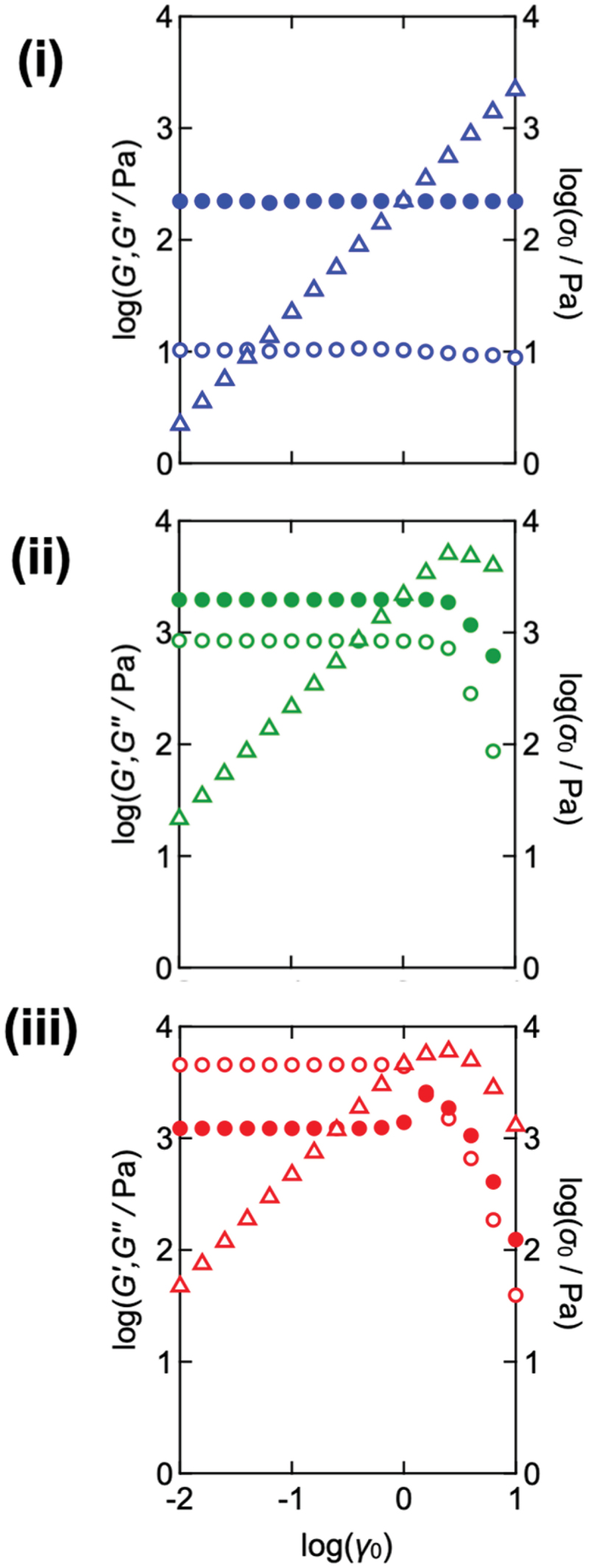
Shear strain (*γ*_0_) dependencies of the storage modulus *G’* (open circles), loss modulus *G”* (filled circles), and shear stress *σ*_0_ (triangles) of the Tetra-PEG slime (*M*_w_ = 20 kg mol^−1^ and *c* = 60 g L^−1^) at various angular frequencies (*ω*). (i) *ω* = 0.1 rad·s^−1^, (ii) *ω* = 1.0 rad·s^−1^, and (iii) *ω* = 10 rad·s^−1^.

Figure 3 shows the results of strain amplitude sweep measurements conducted under various frequencies (*ω*) using strain amplitude (*γ*_0_) as a variable. The shear stress amplitude (*σ*_0_) is also plotted as triangles. At *ω* = 0.1 rad s^−1^, *G'* and *G''* are independent of the applied strain, and *σ*_0_ is linearly proportional to *γ*_0_, which suggests the linear viscoelastic regime. At higher frequencies (*ω* = 1.0 and 10 rad s^−1^), *G'* dropped when *γ*_0_ exceeded the upper limit of the linear regime (*γ*_c_), and *G'* and *G''* became appreciably dependent on *γ*_0_. In principle, it is not possible to determine where the nonlinearity starts to appear. In this paper, *γ*_c_, also referred to as the critical strain amplitude, was regarded as a reference of the onset, and set as the strain amplitude at the data point immediately preceding that at which the measured stress amplitude deviated by more than 5% from the linear fit to the stress – strain response. The strain dependencies of *G'* and *G''* were observed more clearly at higher frequencies and became detectable at smaller strain amplitudes as the frequency increased. This tendency has been observed across various materials, including suspensions, soft gels, and food products [51–53]. The detailed *G'* and *G''* values as a function of strain for the Tetra-PEG slimes with other concentrations and network strand length are shown in Section 1 of the Supporting Information. Notably, in the nonlinear regime, the quantities *G'* and *G''* obtained under LAOS should be regarded as apparent moduli. In this study, they are used only to identify the onset of nonlinearity, while stress waveforms and Lissajous curves confirm the validity of the sinusoidal response within the relevant strain range in the next discussion.

**Figure 4.**
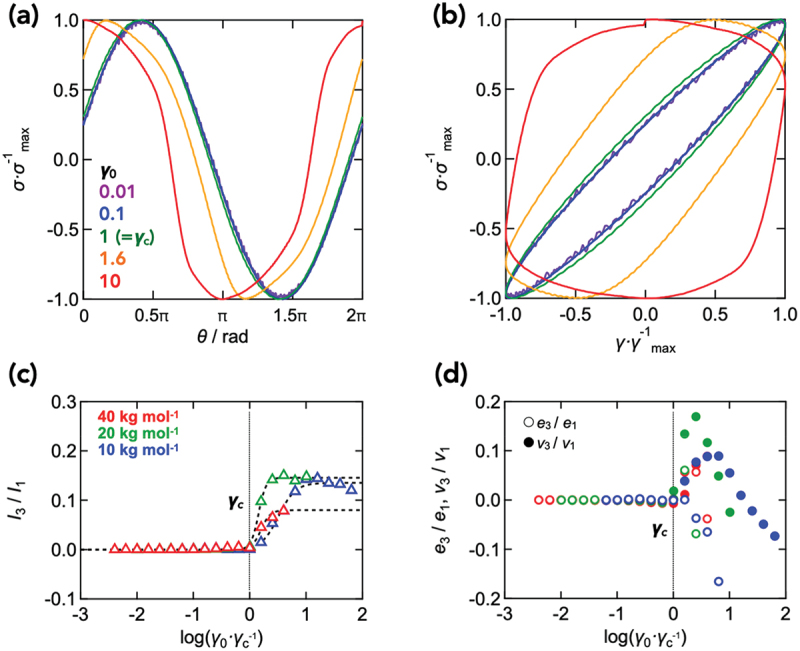
Stress waveform analysis during strain amplitude sweep measurements under various strain amplitudes at an angular frequency of 10 rad s^−1^. Normalized stress as a function of phase of applied strain (a) and normalized applied strain (b) of Tetra-PEG slime (*M*_w_ = 20 kg mol^−1^, *c* = 60 g L^−1^). Harmonics ratio *I*_3_/*I*_1_ (c) and elastic (*e*_3_/*e*_1_) and viscous (*v*_3_/*v*_1_) Chebyshev coefficients (d) as functions of strain normalized against *γ*_c_ of Tetra-PEG slime (*M*_w_ = 10, 20, 40 kg mol^−1^, *c* = 60 g L^−1^).

Figure 4 shows the stress waveform analysis of the Tetra-PEG slime (*M*_w_ = 20 kg mol^−1^, *c* = 60 g L^−1^) under strain amplitude sweep measurements at various strain amplitudes. Up to the critical strain amplitude *γ*_c_, the Lissajous curves remained nearly perfect ellipses, whereas above *γ*_c_ they became progressively distorted, indicating the onset of nonlinear viscoelasticity. This transition was also evident in the harmonics ratio (*I*_3_/*I*_1_), elastic (*e*_3_/*e*_1_) and viscous (*v*_3_/*v*_1_) Chebyshev coefficients, which remained negligible when *γ*_0_ ≤ *γ*_c_ and drastically increased once *γ*_0_ exceeded *γ*_c_. Notably, when the data points for these higher-harmonic measures were subjected to sigmoidal fits, the *γ*_0_-axis intercept of the tangent at the 50% point of the sigmoid fit agreed well with the *γ*_c_. Specifically, *γ*_c_ was defined as the strain amplitude at which *σ*_0_ deviates by 5% from the linear scaling (*σ*_0_ ∝ *γ*_0_), and the onset strain estimated from the maximum-slope tangent of *I*_3_/*I*_1_ fitted with $A\left[1-\frac{1}{1+{\left(\gamma /\gamma_{50\%}\right)}^{\alpha }}\right]$ agree within 10%. Here, *A* is a scaling constant, *γ*_50%_ is the strain amplitude at the 50% point, and *α* controls the sharpness of the transition. This agreement supports the validity of determining *γ*_c_ based on the deviation of *σ*_0_ from the expected proportionality to *γ*_0_.

**Figure 5.**
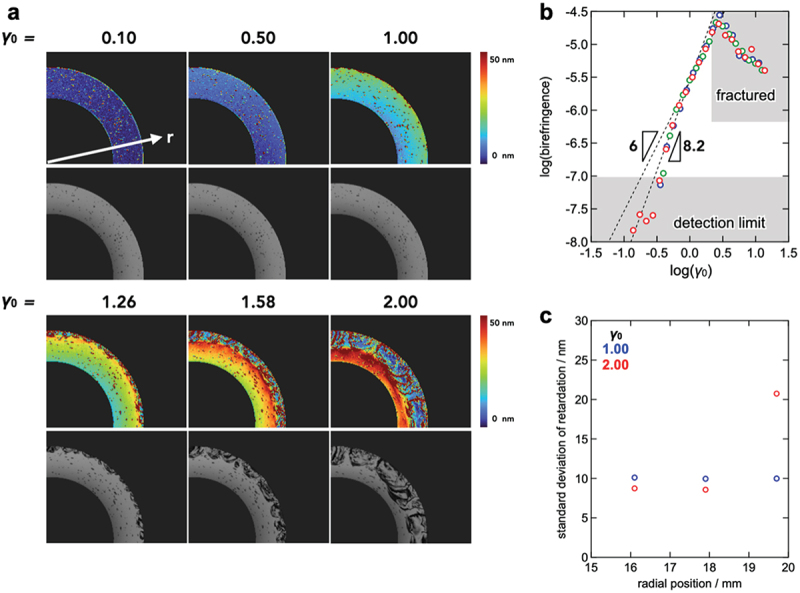
(a) Spatial distribution of the retardation of the Tetra-PEG slime (*M*_w_ = 20 kg mol^−1^, *c* = 60 g L^−1^) under various shear strain amplitudes (*γ*_0_ = 0.10, 0.50, 1.00, 1.26, 1.58, and 2.00). The monochrome images in the lower rows represent the brightness value maps. (b) Birefringence (retardation normalized against thickness) plotted against the strain amplitudes normalized against the radius *r* (blue: *r* = 16.1 mm, red: *r* = 17.9 mm, and green: *r* = 19.7 mm). (c) Radial position dependence of standard deviation of retardation under different strain amplitudes (blue: *γ*_0_ = 1.00, red: *γ*_0_ = 2.00).

We employed rheo-PI to visualize the spatial distribution of birefringence and the structural evolution during the strain-amplitude sweep measurements. Notably, in this study, birefringence was used to assess whether spatially heterogeneous macroscopic orientation develops, and not to perform a full nonlinear optical decomposition under strain-amplitude sweep. Figure 5(a) shows the spatial retardation distribution obtained from the strain-amplitude sweep measurement; here, *r* represents the radial distance from the center of the parallel-plates fixture. The colored regions in the image correspond to areas of active flow within the sample. The dark regions observed at lower *r* values are attributed to light obstruction caused by the shaft of the fixture, while those at higher *r* values indicate regions outside the sample.

It should be noted that, while coaxial cylinder and cone-plate geometries are suitable in principle for determining the stress, the parallel-plate geometry does not rigorously provide correct stress values. Therefore, for the Rheo-PI measurements with the parallel- plates geometry, we restricted the analysis to retardation and did not use the parallel-plate data for conducting quantitative stress-based comparisons across geometries. After applying a correction for strain differences caused by variations in *r*, the retardation was analyzed as a function of the local strain amplitude. The details of this correction are provided in Section 2 of the Supporting Information.

In the linear regime, we evaluated the birefringence by taking the radial average of the retardation at the peak of each oscillation cycle. This peak retardation increased homogeneously with increasing strain (Figure 5(b)). Figure 5(c) shows the radial dependence of the standard deviation of the retardation at the strain maxima for different strain amplitudes. The standard deviation remained small and nearly uniform in the linear regime, but increased markedly near the edges at higher strains, indicating that the sample became increasingly heterogeneous from the outer region. A similar trend was observed in the brightness images. Although the retardation was averaged along the thickness direction, Rheo-PI provided an in-plane spatial map; hence, the radial profiles and the spatial standard deviation of retardation quantified the degree of macroscopic deformation inhomogeneity within the field of view. This implies that the material deformed spatially homogeneously at least up to *γ*_c_, so that the onset of nonlinear behavior from *γ*_c_ was not accompanied by a detectable macroscopic deformation inhomogeneity in the flow and orientation field within the observation plane. Consistent with this, the retardation – strain relation exhibited a change in scaling behavior at around *γ*_c_ (Figure 5(b)), suggesting that the onset of nonlinearity can be tracked optically even when quantitative stress comparison across geometries is not feasible.

In the Rheo-PI experiment, the incident light propagated along the velocity-gradient direction, perpendicular to the shear plane. The measurement thus probed the path-averaged optical anisotropy in the flow – vorticity plane. Nevertheless, the heterogeneous flow behavior could be accessed.

**Figure 6.**
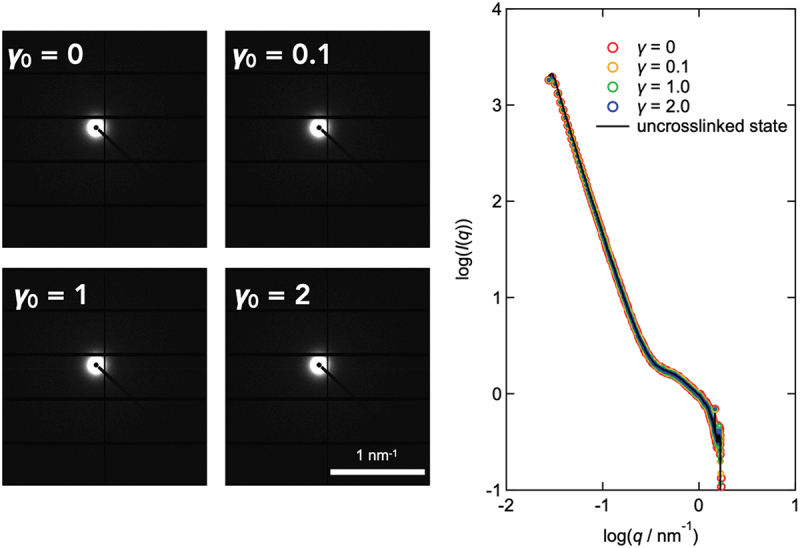
(Left) Rheo-SAXS images of Tetra-PEG slime under various shear strain amplitudes (*γ*_0_). (right) SAXS intensity (*I*) as a function of scattering vector (*q*). The sample was the Tetra-PEG slime with *M*_w_ = 20 kg mol^−1^ and *c* = 60 g L^−1^. The dashed line represents the SAXS intensity of the sample in the uncrosslinked state.

Rheo-SAXS measurements were performed to discuss the microscopic molecular-level changes caused by a larger deformation. Figure 6 shows the SAXS image obtained during strain-amplitude sweep measurements. The SAXS profiles were virtually identical to those of the uncrosslinked tetra-PEG solution (dashed line) within the accessible *q*-range and experimental sensitivity. This suggests that there was no excess scattering in the SAXS range. These results confirm that the characteristic length scales and morphological form factors were unchanged under strain-amplitude sweep measurements, and no shear-induced structural differences were observed. It should be noted that owing to the X-ray beam propagating along the velocity-gradient axis, scattering pattern results sampled material properties only in the flow – velocity-gradient plane, leading to anisotropic scattering patterns due to network strand deformation toward the flow direction under macroscopic deformation. In contrast, the experimental results remained unchanged, exhibiting similar behavior to reported tensile SANS measurements of Tetra-PEG gels [54]. These SAXS and PI results suggest that while the orientational anisotropy appeared larger than the submicron order, the nanostructure of the network remained essentially unaltered. It should be noted that the weak submicron-scale chain orientation detected by birefringence is not expected to generate detectable anisotropy in SAXS, as SAXS probes nanometer-scale electron-density contrast, and the polymer concentrations in our samples were too low for such small deformations to be observed.

**Figure 7.**
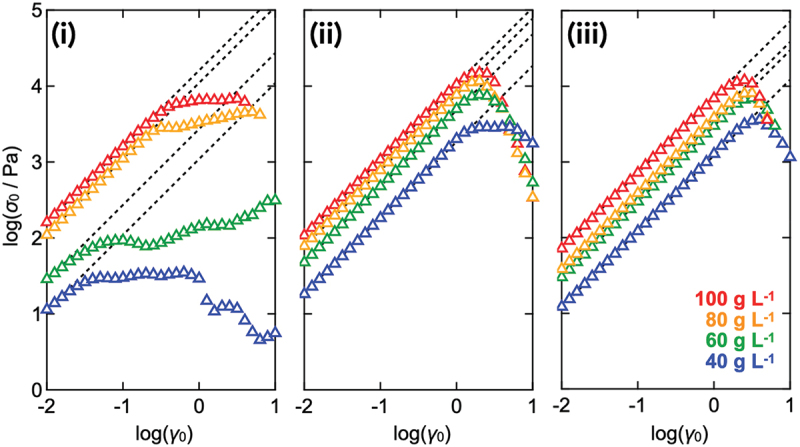
Dependence of the shear stress (*σ*_0_) on shear strain amplitude (*γ*_0_) for Tetra-PEG slimes (i: *M*_*w*_ = 10 kg mol^−1^, ii: *M*_*w*_ = 20 kg mol^−1^, and iii: *M*_*w*_ = 40 kg mol^−1^) with various polymer concentrations, as shown in the legend. The dashed line shows the line-fitting of *σ*_0_ in the linear regime.

Thus far, it has been demonstrated that the onset of nonlinear behavior under strain-amplitude sweep is not governed by microscopic structure formation or the emergence of flow heterogeneity. We thus investigated how the onset of the nonlinear response varied with changes in the network structure to gain further insight into its origin. Figure 7 presents the shear stress amplitude (*σ*_0_) as a function of the shear strain amplitude (*γ*_0_) under strain-amplitude sweep measurements for Tetra-PEG slimes with various molecular weights (*M*_w_ = 10, 20, and 40 kg mol^−1^) and polymer concentrations (*c* = 40–100 g L^−1^). The *σ*_0_ was proportional to *γ*_0_ in the linear regime, but eventually dropped when *γ* exceeded the upper limit *γ*_c_. Further, *σ*_0_ increased with both increasing polymer concentration and decreasing molecular weight, that is, with increasing molar concentrations of the precursors. At low concentrations, slimes with a molecular weight of 10 kg mol^−1^ exhibited nonlinear behavior at smaller strain amplitudes compared to those with molecular weights of 20 and 40 kg mol^−1^.

**Figure 8.**
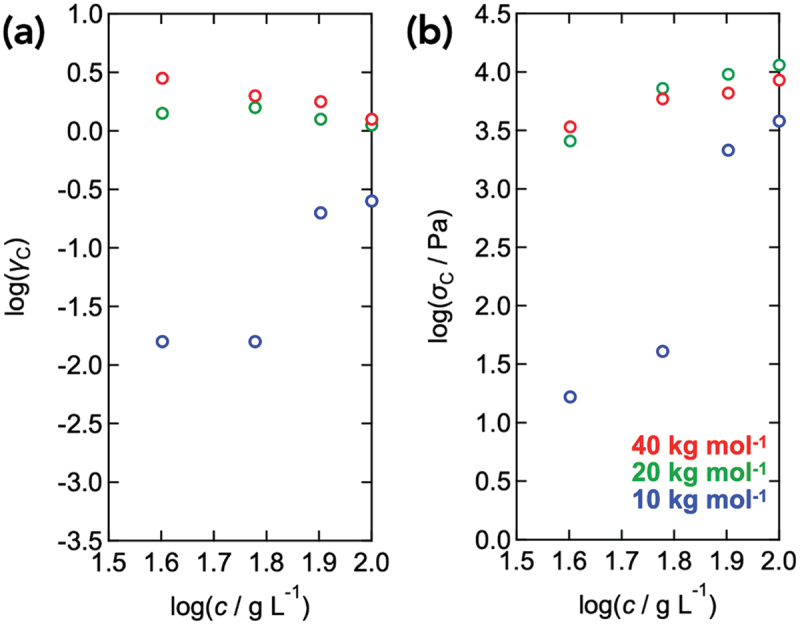
*γ*_c_ (a) and *σ*_c_ (b) against polymer concentration (*c*). The colors represent molecular weights (red: 40 kg mol^−1^, green: 20 kg mol^−1^, and blue: 10 kg mol^−1^).

We define the critical strain (*γ*_c_) and stress (*σ*_c_) amplitude as the linear response between *σ*_0_ and *γ*_0_. Figure 8 represents the values of *γ*_c_ and *σ*_c_ as a function of polymer concentration for each molecular weight (10, 20, and 40 kg mol^−1^). The *γ*_c_ values for the Tetra-PEG slimes with molecular weights of 20 and 40 kg mol^−1^ slightly decreased with increasing *c*, indicating that dense networks show strong nonlinearity at smaller strains. In contrast, for the Tetra-PEG slimes with a molecular weight of 10 kg mol^−1^, the *γ*_c_ exhibited an increase in *γ*_c_ with *c*. Meanwhile *σ*_c_ increased with *c* for all samples. Tetra-PEG slimes with molecular weights of 20 and 40 kg mol^−1^ showed similar absolute values, whereas the 10 kg mol^−1^ samples exhibited slightly lower values. All samples did not show a clear power law.

Previous studies on various soft materials have shown that the critical strain amplitude *γ*_c_ can be relatively insensitive to polymer concentration, whereas the corresponding characteristic stress exhibits clear polymer concentration dependence. These observations support the view that the onset of nonlinearity is primarily governed by short-range interparticle interactions rather than changes in the internal network elasticity [55–57]. However, our results do not conform to either of these scaling behaviors: neither a concentration-independent *γ*_c_ nor a clear *σ*_c_ ∝ *c*^2^ relationship was observed. This deviation indicates that the nonlinear response of the Tetra-PEG slimes originates from a distinct mechanism.

For further discussion, we investigated the maximum cycle-integrated elastic contribution per network strand at the critical strain (*W*_c,0_). According to the elastic theory [58,59], *W*_c,0_ can be expressed as

(4) $$\begin{array}{c} W_{c,0}=\frac{1}{2}\frac{{G{\;}^{\prime }}_{c}\gamma_{c}^{2}}{\frac{c}{M_{w}}} \end{array}$$

**Figure 9.**
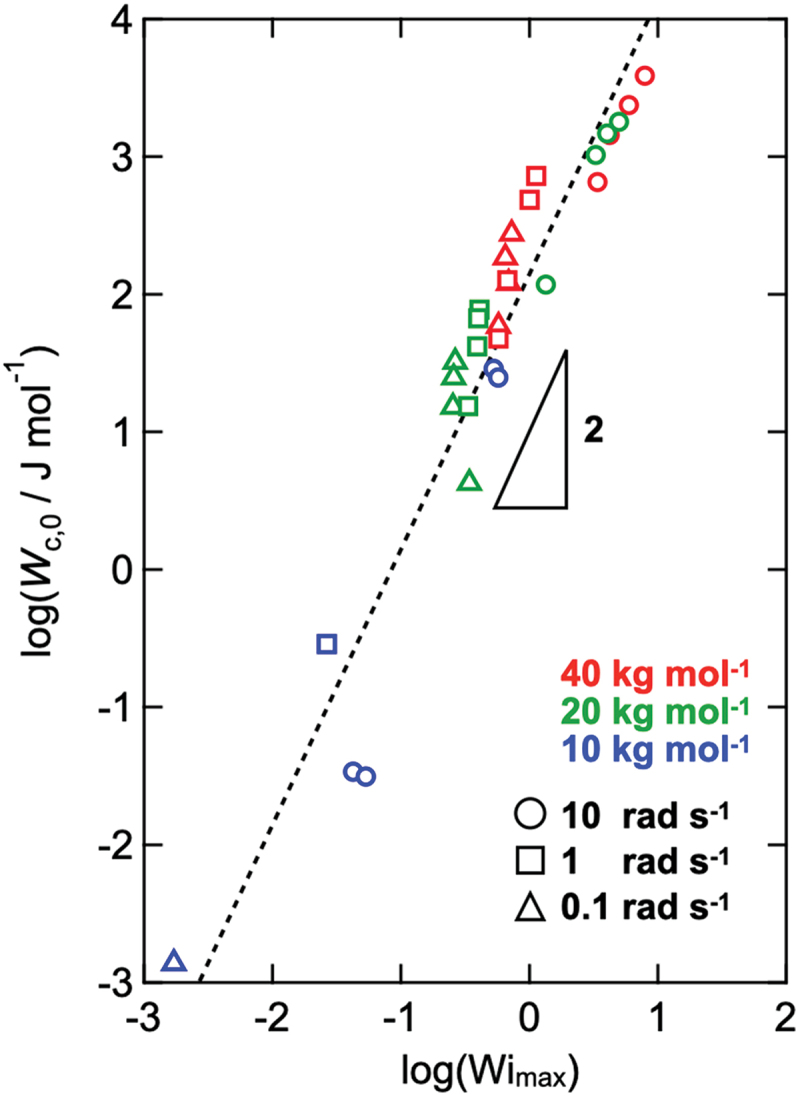
Maximum cycle-integrated elastic contribution per network strand at the critical strain (*W*_c,0_) against Weissenberg number (Wi_max_). The colors represent molecular weight (red: 40 kg mol^−1^, green: 20 kg mol^−1^, and blue: 10 kg mol^−1^), and the symbols represent the frequency (circle: 10 rad s^−1^, square: 1 rad s^−1^, 0.1 rad s^−1^).

Figure 9 presents the relationship between *W*_c,0_ and the maximum value of the Weissenberg number (Wi_max_), which is the shear strain rate normalized by the terminal relaxation time, obtained via strain-amplitude sweep measurements [44]. Here, the applied strain was expressed as *γ* = *γ*_0_sin (*ωt*). Wi_max_ is expressed as

(5) $$\begin{array}{c} Wi_{\max}=\gamma_{c}\omega \tau \end{array}$$

where *τ* represents the terminal relaxation time obtained from the SAOS measurements. The data for all the Tetra-PEG slimes approximately collapsed onto a master curve showing the relationship *W*_c,0_ ∝ Wi_max_^2^, which indicates that the elastic contribution per network strand at the onset of nonlinearity was governed by the balance between deformation and relaxation, as captured by the Weissenberg number. Because both Δ*G* and *τ* differed among samples, normalization against *W*_c,0_ (energy per strand) and Wi_max_ was required to allow meaningful comparison of the onset strain across compositions.

It should be noted that both *W*_c,0_ and Wi_max_ depend on the linear viscoelastic parameters *ΔG* and *τ*. In Eq. (4), the storage modulus *G'*_c_ at the strain amplitude sweep frequency can be expressed using a Maxwell-type description. In Eq. (5), the terminal relaxation time *τ* was introduced directly through Wi_max_ = *γ*_c_ *ωτ*. Remarkably, the exponent, that is, the fitted slope, is close to 2 (although some scatter remains), coinciding with the *σ*_c_ ∝ *c*^2^ relationship reported for cellulose fiber dispersions.

However, we interpret this scaling only as an empirical trend, and note that this comparison is intended only to highlight a similarity in the scaling exponent, not to imply any microscopic or mechanistic correspondence between transient networks and fiber dispersions. Here, both the per-strand energy and Wi_max_ should be viewed as empirical normalization parameters to organize the onset data, not as full descriptors of the nonlinear elastic response. Taken together, these observations indicate that nonlinearity emerges when the elastic contribution per strand reaches a characteristic scale, without implying a universal mechanism or a direct correspondence with fiber-network yielding.

## Conclusion

We investigated the effects of network structure on the nonlinear behavior of a model transient network (Tetra-PEG slime) under strain-amplitude sweep measurements. The key findings are summarized as follows: (1) strain-amplitude sweep measurements revealed a distinct transition from linear to nonlinear behavior, accompanied by a reduction in modulus and deviation from proportionality between stress and strain; (2) Rheo-PI confirmed that the Tetra-PEG slime deformed homogeneously in the linear regime, while fracture-like disturbances appeared at its edges under nonlinear conditions; (3) Rheo-SAXS measurements showed no emergence of structural changes at the length scale detectable by SAXS, even in the nonlinear regime; and (4) elastic contribution per network strand at the critical strain collapsed onto a single master curve when plotted against the Weissenberg number, following *W*_c,0_ ∝ Wi_max_^2^. This scaling suggests that the onset of nonlinear behavior is governed by the balance between molecular relaxation and applied deformation, and that nonlinear behavior occurred when the elastic contribution per strand reached a critical threshold determined by short-range strand – strand interactions.

These findings help establish a quantitative framework for understanding nonlinear viscoelasticity in transient networks and provide new insights into the molecular origins of nonlinearity in soft matter.

## Supplementary Material

Supplemental Material
