# Supplementary material for: Strain amplitude sweep testing of oscillatory shear of transient networks with controlled network structures
Source: Sci Technol Adv Mater. 2026 Jan 12;27(1):2613918. doi: 10.1080/14686996.2026.2613918 (PMC12934341; doi:10.1080/14686996.2026.2613918)
Supplement: Supplemental Material [file TSTA_A_2613918_SM8067.pdf]

Supporting Information: Strain amplitude sweep testing of oscillatory shear behavior of transient networks with controlled network structures

Ren Sato[a], Yoshifumi Yamagata[b,c], Moe Araida[a,b], Taisuke Sato[d], Mitsuru Naito[e], Hiroshi Sekiguchi[f], Keishi Akada[f], Ung-il Chung[a,g], Takuya Katashima[a,*]

[a]*Department of Bioengineering, Graduate School of Engineering, The University of Tokyo, 7-3-1 Hongo, Bunkyo-ku, Tokyo 113-8656, Japan;* [b] *Anton Paar Japan K.K., Riverside Sumida 1st Fl, 1-19-9, Tsutsumi-dori, Sumida-ku, Tokyo, 131-0034, Japan;* [c] *Research Institute for Science and Technology, Tokyo University of Science, 2641 Yamazaki, Noda-shi, Chiba, 278-8510, Japan;* [d] *Photonic Lattice Inc., LABO CITY SENDAI, 6-6-3 Minami-Yoshinari, Aoba-ku, Sendai-city, Miyagi, 989-3204, Japan;*

[e]*Department of Materials Science and Technology, Faculty of Advanced Engineering, Tokyo University of Science, 6-3-1, Niijuku, Katsushika-Ku, Tokyo 125-8585, Japan;*

[f] *Japan Synchrotron Radiation Research Institute, 1-1-1, Kouto, Sayo-cho, Sayo-gun, Hyogo, 679-5198 Japan;* [g] *Center for Disease Biology and Integrative Medicine, Graduate School of Medicine, The University of Tokyo, 7-3-1, Hongo, Bunkyo-ku, Tokyo 113-8656, Japan*

* *To whom correspondence should be addressed:* katashima@g.ecc.u-tokyo.ac.jp (T.K.);
TEL: +81-3-5841-1876 (T.K.)

Section 1. Strain dependence of storage and loss moduli of Tetra-PEG slime.

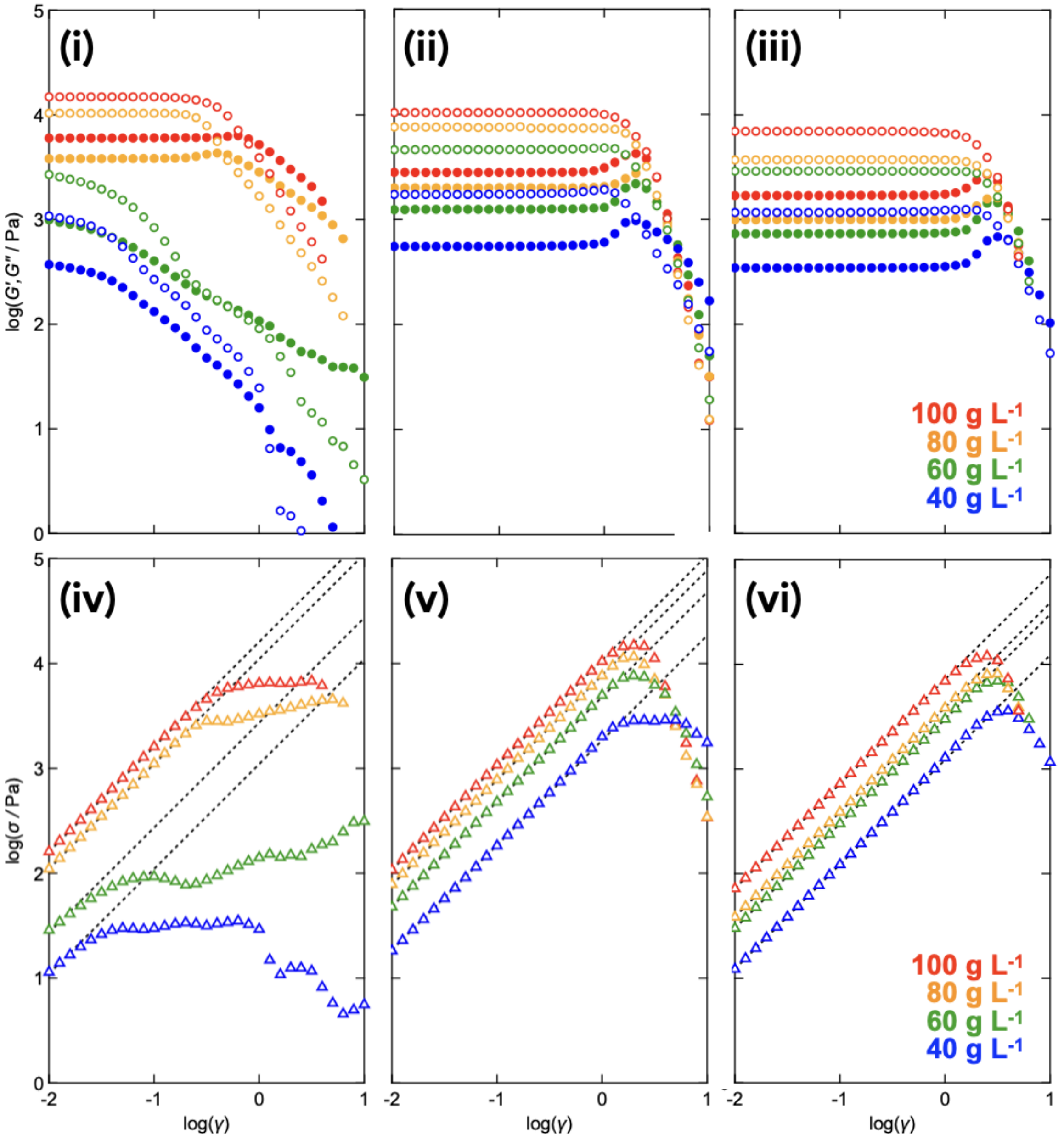

Figure S2. Strain dependence of the storage (G') and loss (G'') moduli (top) and stress (σ) (bottom) of the Tetra-PEG slimes (i, iv: M = 10 kg mol^{-1}, ii, v: M = 20 kg mol^{-1}, iii, vi: M = 40 kg mol^{-1}) with various polymer concentrations. The dashed line shows the line-fitting of σ in the linear regime.

Section 2. Correction for radial strain variation in the parallel-plate geometry

In the parallel-plate geometry, the reference radial distance (r_0) is defined at two-thirds of the plate radius (R) for the MCR 302 rheometer. The true strain amplitude is proportional to the radial distance (r). Accordingly, the true strain at any radial distance can be expressed as:

$$\gamma = \gamma_{\text{app}} \frac{r}{r_0} \tag{S1}$$

Here, γ denotes the strain corrected for the radial distance, whereas γ_{app} represents the apparent strain amplitude defined in the rheometer.

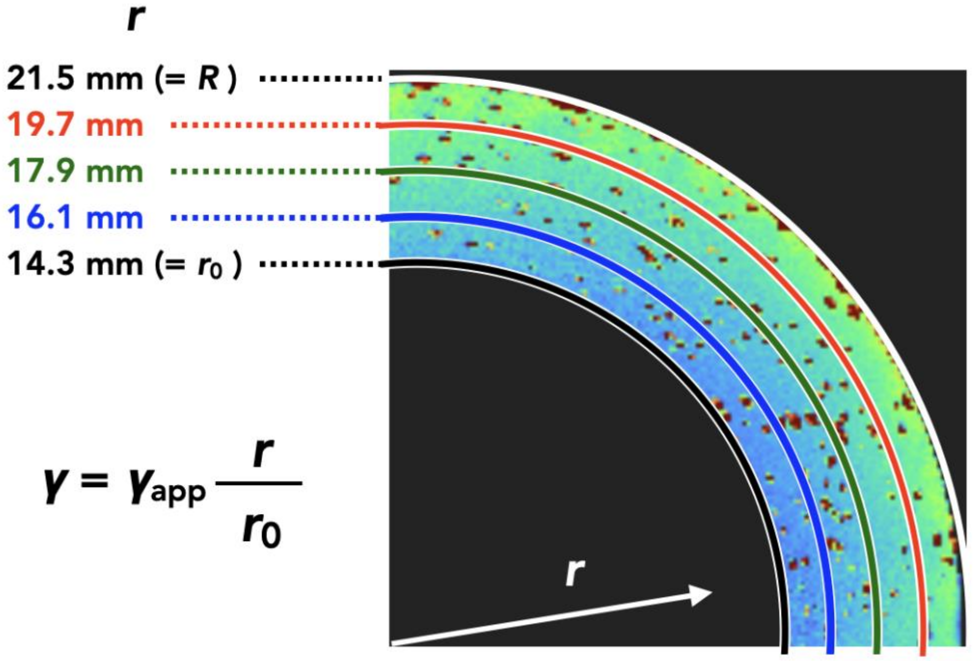

Figure S3. Apparent shear strain and retardation of tetra-PEG slime under strain amplitude sweep measurements plotted against time, measured at different radial distances (black: $r = r_0 = 14.3$ mm, blue: $r = 16.1$ mm, green: $r = 17.9$ mm, red: $r = 19.7$ mm).

Birefringence values were extracted from three radial distances, as indicated by the colored lines in Figure S1. Figure S2 presents the birefringence plotted as a function of the apparent

strain (γ_{app}) and the true strain (γ). When plotted against γ_{app}, the data obtained at different radial distances exhibited a noticeable broadening and did not fully overlap. In contrast, when plotted against the corrected strain γ, the birefringence values collapsed onto a single curve and showed good agreement up to approximately $\gamma \approx 2$. At higher strain amplitudes, deviations became evident, which are attributed to the onset of sample fracture.

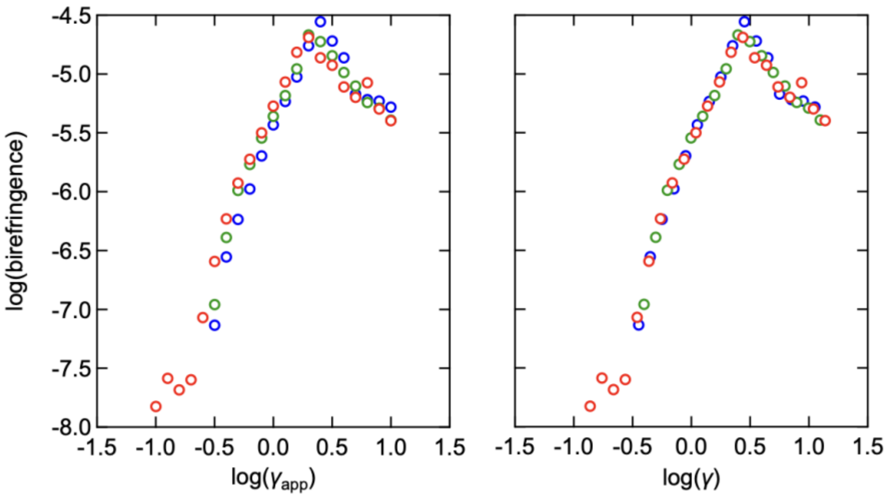

Figure S4. Birefringence (retardation normalized against thickness) plotted against the apparent strain (left) and true strain (right) (blue: r = 16.1 mm, green: r = 17.9 mm, red: r = 19.7 mm).
